# Genome Size Covaries More Positively with Propagule Size than Adult Size: New Insights into an Old Problem

**DOI:** 10.3390/biology10040270

**Published:** 2021-03-26

**Authors:** Douglas S. Glazier

**Affiliations:** Department of Biology, Juniata College, Huntingdon, PA 16652, USA; glazier@juniata.edu

**Keywords:** allometric scaling, cell size, cellular (nuclear) DNA content, Crustacea, egg and sperm sizes, life cycles, multicellular animals and plants, nucleotypic effects, spore, pollen and seed sizes, unicellular organisms

## Abstract

**Simple Summary:**

The amount of hereditary information (DNA) contained in the cell nuclei of larger or more complex organisms is often no greater than that of smaller or simpler organisms. Why this is so is an evolutionary mystery. Here, I show that the amount of DNA per cell nucleus (‘genome size’) relates more positively to egg size than body size in crustaceans (including shrimp, lobsters and crabs). Genome size also seems to relate more to the size of eggs or other gametes and reproductive propagules (e.g., sperm, spores, pollen and seeds) than to adult size in other animals and plants. I explain these patterns as being the result of genome size relating more to cell size (including that of single-celled eggs) than the number of cells in a body. Since most organisms begin life as single cells or propagules with relatively few cells, propagule size may importantly affect or be affected by genome size regardless of body size. Relationships between genome size and body size should thus become weaker as body size (and the amount of cell multiplication required during development) increases, as observed in crustaceans and other kinds of organisms.

**Abstract:**

The body size and (or) complexity of organisms is not uniformly related to the amount of genetic material (DNA) contained in each of their cell nuclei (‘genome size’). This surprising mismatch between the physical structure of organisms and their underlying genetic information appears to relate to variable accumulation of repetitive DNA sequences, but why this variation has evolved is little understood. Here, I show that genome size correlates more positively with egg size than adult size in crustaceans. I explain this and comparable patterns observed in other kinds of animals and plants as resulting from genome size relating strongly to cell size in most organisms, which should also apply to single-celled eggs and other reproductive propagules with relatively few cells that are pivotal first steps in their lives. However, since body size results from growth in cell size or number or both, it relates to genome size in diverse ways. Relationships between genome size and body size should be especially weak in large organisms whose size relates more to cell multiplication than to cell enlargement, as is generally observed. The ubiquitous single-cell ‘bottleneck’ of life cycles may affect both genome size and composition, and via both informational (genotypic) and non-informational (nucleotypic) effects, many other properties of multicellular organisms (e.g., rates of growth and metabolism) that have both theoretical and practical significance.

## 1. Introduction

Two fundamental properties of all living systems are their physical size (‘body size’) and the quantity of their genetic material (DNA) per cell (‘genome size’, which refers to either the haploid or total DNA content per cell nucleus: see [1] for a review of this term). Numerous biological and ecological traits relate to body size [2,3,4,5,6] and genome size [7,8,9,10,11,12]. At first thought (and without further knowledge), one might think that the body size and genome size of organisms, i.e., the magnitudes of their phenotype (physical structure) and genotype (DNA information), should be strongly related. It seems reasonable to assume that more genetic information should be required to build larger (often more complex) organisms than smaller ones.

However, genome size appears to be unrelated (or only weakly related) to organismal complexity, apparently (at least in part) because much of the DNA in the genome does not consist of genes that code for RNA and proteins making up the structure of the body [7,8,13,14,15,16] (but see [17,18]). Much of the DNA in eukaryotic organisms consists of replicated sequences, which can vary greatly in quantity independently of the size or complexity of an organism [7,8,13,14,15,16]. The existence of replicated DNA helps explain why genome size is not necessarily related to body size or complexity, the so-called ‘C-value paradox’; but why the quantity of replicated DNA has evolved to be so different among species, is still little understood [7,8,14,15]. Although the proximate mechanisms involved are quite well understood (e.g., mobile or transposable DNA and polyploidy are importantly involved in genome expansion [8,9,14]), the ultimate (evolutionary) causes of genome-size variation remain unclear. Another related mystery is why the body-size scaling of genome size is highly diverse taxonomically, showing positive (strong or weak) relationships in many taxa, but no or even negative relationships in many others (Table 1). The primary aim of my article is to try to help explain this surprising diversity of relationships between genome size and body size. I hope that my exploratory analyses will stimulate others to investigate further the functional mechanisms and evolutionary causes underlying this diversity of genome-size scaling.

Crustaceans are an excellent taxonomic group for studying the body-size scaling of genome size because (1) they encompass a broad range of body sizes (>nine orders of magnitude in body mass [101]), (2) the genome size of many (>400) species has been determined [102], and (3) crustacean taxa show diverse genome sizes (nearly 650-fold [62]) and body-size scaling relationships [57,103], thus providing a useful model system for exploring the causes of genome-size diversity.

In this article, I explore whether crustacean genome size correlates more strongly with egg size than adult size. This objective was motivated by the remarkable similarity between the body-size scaling of genome size in various crustacean taxa [57,103] and that observed for egg size in the same taxa [101], as further described in the Results (Section 3). As will be seen, crustacean genome size does correlate more strongly with egg size than adult size, and this pattern can be explained in terms of (1) single-celled eggs being a critical first step in all animal life histories, and (2) the typically strong relationship observed between genome size and cell size. I further suggest that the body-size scaling of crustacean genome size varies considerably because (1) genome size relates more strongly to cell size (including egg size) than to the number of cells in a multicellular body, and (2) the proportional effects of cell size and number on body size vary greatly among taxa (also see [44,64]). This perspective provides insight into the causes of variation in genome size and its relationship to organismal size, as I further illustrate with applications to other animal and plant taxa. I also promote the view that biological scaling analyses should be expanded beyond the traditional focus on adult size to include the sizes of other developmental stages, as well.

## 2. Materials and Methods

### 2.1. Data Sources

I obtained data on genome size (haploid DNA content per cell nucleus, pg) and maximum body length (mm) for 170 species of four major taxa of crustaceans (Cladocera, Copepoda, Peracarida and Decapoda) from the supplementary information in [57]). For comparison, I also used data in [101] on egg mass (mg) and adult (maternal) body mass (mg) for 262 species in the same four taxa as above. Additional genome-size data from various tissues (including exopodites, gills, testes, haemocytes, coelomocytes, muscle cells, heart cells, and whole-body samples) of various crustacean species were collected from [102]. Data on body mass, egg mass and genome size are available in Table S1.

### 2.2. Scaling Analyses

I scaled genome size versus egg mass or adult mass or length using least squares regression of log_10_-tranformed values, so as to linearize and normalize the data, and to permit proportional relationships to be readily discerned (following [104,105]). I also used general linear model (GLM) analyses to compare the relative strength of relationships between genome size and egg versus adult size. I used SYSTAT 10 software (SPSS Inc., Chicago, IL, USA) for all statistical analyses.

## 3. Results

**Figure 1:**
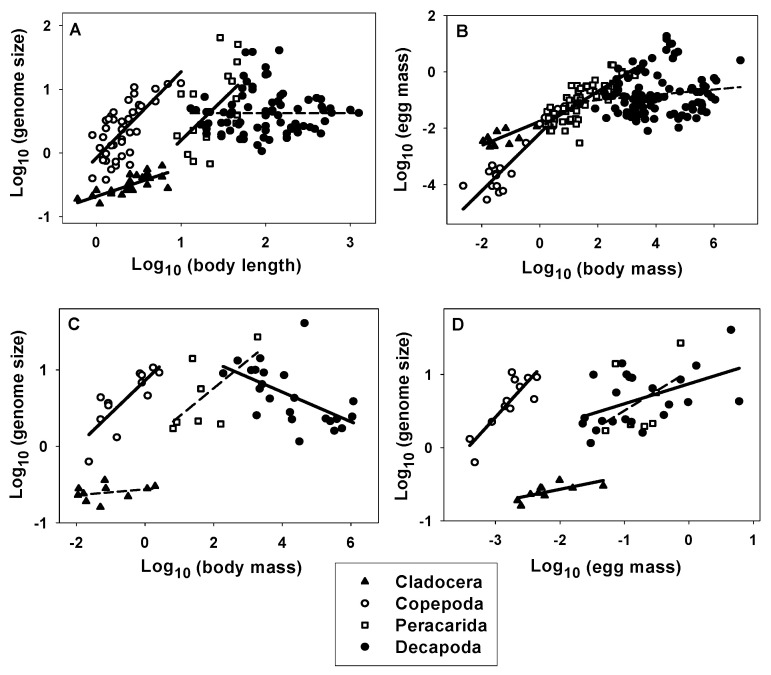
Log-linear relationships between genome size (pg) and body length (mm) (**A**: data from [57]), wet egg mass (mg) and wet adult (maternal) body mass (mg) (**B**: data from [101]), genome size and wet body mass (**C**: data from [101,102]), and genome size and wet egg mass (**D**: data from [101,102]) for four major crustacean taxa. Solid and dashed lines indicate significant and non-significant linear regressions, respectively (details in Table 2).

Relatively parallel scaling exponents (slopes) occur between the relationships of genome size with body length and of egg mass with body mass among the four major crustacean taxa sampled (Figure 1A,B; Table 2). For both kinds of relationships, the slopes decrease in the same order: Copepoda, Peracarida, Cladocera and Decapoda (Table 2). These and similar differences in the scaling of genome size with body mass among these four taxa (Figure 1C; Table 2) suggested that genome size should be positively correlated with egg mass, which was confirmed (Figure 1D; Table 2). The greater positive effect of egg mass versus body mass on genome size is indicated by the greater scaling slopes of genome size in relation to egg mass than to body mass in all four taxa (Table 2).

A GLM analysis also revealed that in the Cladocera, Copepoda and Decapoda, the effect of egg mass on genome size was significantly positive after controlling for the effect of body mass, whereas the effect of body mass was non-significant or significantly negative after controlling for the effect of egg mass (Table 3). The only exception to this pattern was the Peracarida, which showed no significant effects of egg mass or body mass (after controlling for the other) on genome size, probably because of the small sample size (Table 3).

**Figure 2:**
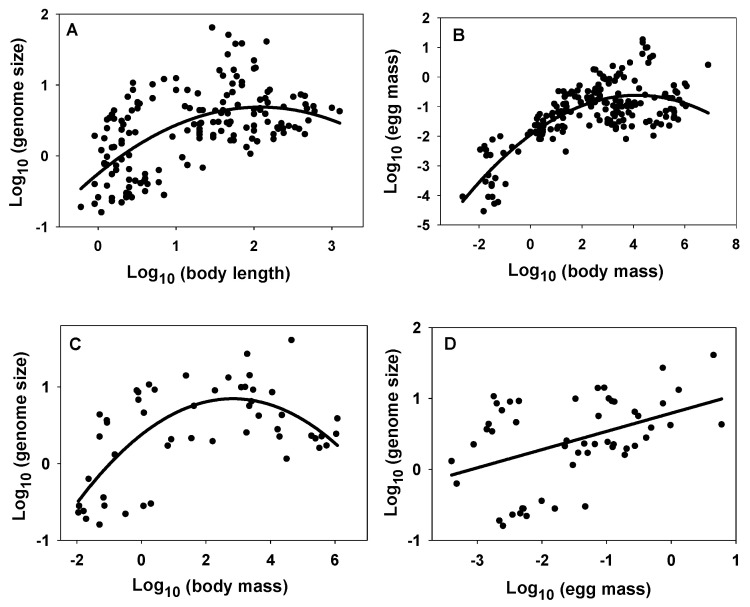
Curvilinear relationships between crustacean genome size (pg) and body length (mm) (**A**), wet egg mass (mg) and wet adult (maternal) body mass (mg) (**B**), and genome size and wet body mass (**C**). Note contrast with linear relationship between genome size and wet egg mass (**D**). All relationships based on log-transformed data in Figure 1 (statistical details in Table 4).

Another pattern emerged when the data for all the sampled crustacean species were scaled together. The relationships between genome size and body length or body mass, and between egg mass and body mass were all significantly curvilinear (concave downward), whereas the relationship between genome size and egg mass was significantly linear (Figure 2; Table 4). These patterns indicate that genome size correlates more positively with egg mass and the body size of relatively small crustaceans than with the body size of relatively large crustaceans.

## 4. Discussion

### 4.1. Scaling of Crustacean Genome Size with Egg versus Adult Body Sizes

The results of this study indicate that crustacean genome size correlates more positively with egg mass than adult body mass. Furthermore, relationships between genome size and body size appear to be stronger in small versus large crustaceans, as revealed by the curvilinear (concave downward) scaling depicted in Figure 2A,C. This trend is consistent with the observation that egg mass also scales curvilinearly (concave downward) with body mass in a similar way (Figure 2B; also see [99]). Since genome size is a linear function of egg mass (Figure 2D), and egg mass relates more positively to body mass in small versus large crustaceans (Figure 2B), it follows that genome size should also relate more positively to body size in small versus large crustaceans, as observed (Figure 2A,C). This difference is highlighted by a comparison of two taxa with the largest sample sizes: microscopic copepods and macroscopic decapods. In copepods, genome size is strongly positively correlated with both egg mass and body size, whereas in decapods, genome size is positively correlated with egg mass, but non-significantly related to body length and negatively related to body mass (Figure 1A,C,D; Table 2 and Table 3). However, in both taxa, egg mass is a stronger positive predictor of genome size than is body mass (Table 3). Before attempting further explanation of these patterns, I discuss next whether they may also apply to reproductive propagules in other organisms.

### 4.2. Scaling of Genome Size with Sizes of Gametes and Propagules in Other Animal and Plant Taxa

I surveyed the literature to investigate whether genome size is more positively related to the size of eggs and other reproductive propagules (spores, pollen and seeds) or gametes (sperm) than to body size in other organisms. Table 5 shows that in various multicellular plants and animals at various taxonomic levels, genome size is frequently positively correlated with propagule size.

Although a crude comparison because of the variation in taxonomic levels represented (from species to phyla or divisions), genome size of multicellular organisms appears to be correlated positively with propagule size (69%: 49/71) much more frequently than with body size (39%: 29/75; Table 1). These suggestive differences deserve to be explored in a more rigorous way, as I have done here for crustaceans.

### 4.3. Single-Cell ‘Bottlenecks’ in the Life Cycles of Multicellular Organisms May Affect Their Genome and Cell Sizes

In this section, I propose the Single-Cell ‘Bottleneck’ Hypothesis (SCBH) to explain why genome size appears to relate more positively to the sizes of eggs and other reproductive propagules than to body size, and why relationships between genome size and body size vary so greatly among different kinds of crustaceans and other organisms (Table 1). The SCBH has eight well-verified assumptions and five testable predictions (Table 6).

Assumption #1 is not only nearly always true [154], but also supported by theory (e.g., [155,156]). As Bonner [154] remarked, the unicellular unfertilized egg “is the minimum unit of inheritance that joins one life cycle to the next. The point of minimum size in the cycle is therefore also the smallest possible unit of heredity” (p. 127). According to multi-level selection theory, single-celled propagules ensure cooperation among the cells of multicellular organisms [155,156,157,158,159,160,161,162]. Development from a single cell minimizes competition among somatic cells because they all receive the same genes, and thus are genetically identical except for somatic mutations [156,157,158]. As Grosberg and Strathmann [157] stated: “If cells have a legislature of lineages like the parliament alleged for genes, then a multicellular organism is a clonal congress. It is the unicellular bottleneck that maintains a voting block of genetically identical cells that is overwhelmingly large.” (p. 115). “With a unicellular bottleneck, defecting cell lineages rarely succeed beyond the life span of the multicellular individual.” (p. 621). This allows evolutionary selection at the individual (cell-group) level to predominate over selection at the cell level [162,163,164,165], which, as I argue in Section 4.4.2, has important consequences for both the size and composition of the genome.

Assumption #2 is common knowledge, based on an enormous amount of histological work.

Assumption #3 is supported by many studies, showing that variation in the sizes of multicellular propagules (e.g., pollen and seeds) is related to variation in cell size (at least in part), both in the propagules themselves and in the somatic body ([109,115,116,166,167,168,169,170,171]; see also Section 4.4; Table A1).

Assumption #4 is supported by numerous data sets in both plants and animals and is universally accepted, at least as a very common rule (e.g., [7,8,9,15,16,19,20,31,116,172,173,174,175,176,177,178,179,180,181,182,183,184,185,186,187,188]).

Assumption #5 is supported by many studies, showing that although increasing genome size (including polyploidy) is almost always associated with increased cell size [179,184,189,190,191,192,193,194,195], it usually has no or a negative relationship with cell number (as indicated by no or only small increases or decreases in body size [179,184,193,195,196,197,198,199,200,201,202,203,204] (see also Section 4.5; Table A2).

Assumption #6 is supported by simple logic. Growth and development of multicellular organisms involve various degrees of cell multiplication and enlargement depending on the kind of organism [103,203,205,206,207].

Assumption #7 is supported by the fact that large organisms tend to grow more by cell multiplication than cell enlargement. In many kinds of multicellular organisms (especially vertebrate animals), body size is only weakly related to cell size (e.g., [3,177,205,208,209,210,211,212,213,214]), thus requiring that increased body size must be largely due to cell multiplication [4,203,211].

Assumption #8 is supported by the common observation that at a given body size, increases in the sizes of somatic cells or reproductive propagules tend to be accompanied by decreases in their number (e.g., [101,146,166,184,196,197,198,199,202,204,215,216,217,218,219,220,221,222]); see also Section 4.4; Table A1).

Prediction #1 (following from assumptions 1–7) is supported by my analyses of crustacean genome size, egg size and body size (Section 3: Figure 1 and Figure 2; Table 2, Table 3 and Table 4), and my overview of relationships of genome size with propagule size and body size in other animals and plants (Section 4.2: Table 1 and Table 5).

**Figure 3:**
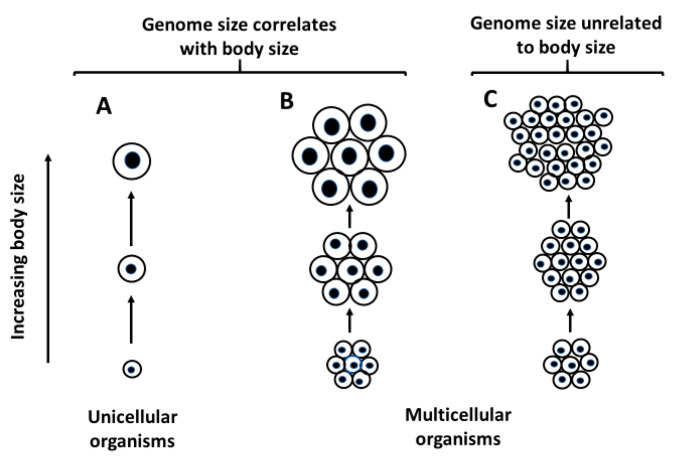
Schematic diagrams illustrating relationships between genome size, cell size and body size in unicellular and multicellular organisms, following predictions #2 and #3 of the Single-Cell ‘Bottleneck’ Hypothesis (SCBH: Table 6). (**A**): Genome size (indicated by the size of the black nucleus in each cell) correlates positively with cell size in unicellular organisms. (**B**): Genome size correlates positively with body size in multicellular organisms that differ largely in cell size. (**C**): Genome size does not correlate with body size in multicellular organisms that differ largely in cell number. Weak correlations between genome size and body size may occur if body size is related to both cell size and number (a situation intermediate between **B** and **C**).

Prediction #2 (as illustrated in Figure 3; and following from assumptions 4–7) is supported by observations in Table 1. Genome size is positively related to body size for all unicellular taxa in my database (100%: 12/12), whereas it is positively correlated with body size much less frequently in multicellular taxa (39%: 29/75) (also see [186]). Interestingly, a strong relationship between genome size and body size is also found in acellular viruses [223].

Prediction #3 (as illustrated in Figure 3; and following from assumptions 4–6) is advocated by [44,57,64,103]. It is especially well supported by a comparison of copepods with decapods. Adult copepods tend to have similar cell numbers regardless of their body size, and thus interpopulation and interspecific variation in body size is strongly related to variation in cell size [44,58,59,224,225]. Therefore, genome size, which is more related to cell size than number, is strongly positively related to adult body size in copepods (Figure 1A,C; Table 2). By contrast, variation in the adult body sizes of decapods appears to be more related to cell number than cell size. In support, haemocyte sizes are similar in decapods varying greatly in adult body size (including shrimp, crayfish, crabs and lobsters [226,227,228,229,230,231]). Accordingly, genome size, a strong indicator of cell size (following assumption #4), is unrelated to body length and somewhat negatively related to body mass in decapods (Figure 1A,C; Table 2). More observations of variation in cell size and number in decapods (and other animals) with different body sizes are needed to further test this prediction.

Prediction #4 (following from assumptions 2–7) is supported by the observation that microscopic copepods show strong positive relationships between genome size and body size, whereas much larger macroscopic decapods do not (Figure 1A,C; Table 2). Furthermore, the curvilinear (concave downward) scaling of genome size with body size in crustaceans, as a whole, is consistent with this prediction. At small body sizes, genome size scales positively with body size, whereas at large body sizes, it is unrelated to or even scales somewhat negatively with body size (Figure 2A,C; Table 4).

**Figure 4:**
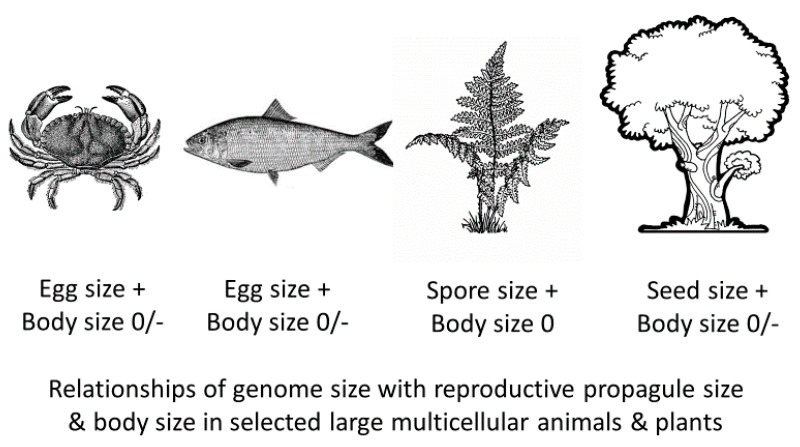
Representative pictures of relatively large multicellular organisms, including decapod crustaceans, bony fishes, ferns and flowering plants [232,233,234,235] that show positive (+) relationships between genome size and reproductive propagule size, but no (0) or weakly negative (−) relationships with adult body size (Table 1 and Table 2), largely following predictions #1, #3 and #4 of the Single-Cell ‘Bottleneck’ Hypothesis (SCBH: Table 6). These relationships occur apparently because genome size is more related to cell size (including the cells of eggs, spores and seeds) than to cell number (which mainly determines the various sizes of relatively large organisms) (following assumptions #2–#5 of the SCBH: Table 6).

Prediction #4 is also consistent with multiple reports that the genome sizes of relatively large animals (e.g., fishes and tetrapods, including huge dinosaurs) and vascular plants (e.g., ferns and flowering plants, including huge trees) tend to show no, or weakly positive or negative relationships with body size (Table 1; Figure 4). As predicted, among mammals, relatively small Rodentia show a weakly positive correlation between genome size and body mass, whereas relatively large Primates, Carnivora and Artiodactyla show no significant relationships (Table 1). However, bats, which include many species at the small end of the mammalian size distribution, may or may not show a significant relationship between genome size and body mass (Table 1).

Although the above results provide significant support for prediction #4 of the SCBH, the great variation in genome-size:body-size relationships shown by various taxa of small-bodied invertebrates is unexpected. Although many studies have reported positive relationships between genome size and body size in small invertebrate taxa (e.g., flatworms, polychaete worms, mollusks, cladocerans, copepods, amphipods, ostracods, mites and ticks, and some rotifers and insect taxa), as predicted, several nonsignificant (or even negative) relationships have also been reported, as well (e.g., nematodes, rotifers, oligochaete worms, spiders, and many insect taxa) (Table 1). Some possible explanations for this surprising variation are provided in [8,57,64] and other sources cited in Table 1. Unfortunately, some of these explanations also appear to be inadequate. For example, it has been suggested that taxa showing determinate growth are more likely to exhibit positive associations between genome size and body size than those exhibiting indeterminate growth [64]. However, existing crustacean data contradict this hypothesis. Although both cladoceran and peracaridan crustaceans exhibit indeterminate (postmaturational) growth, they still exhibit significant associations between genome size and body size, as do copepods and ostracods that show determinate growth (Figure 1A,C; [64,236]). Unfortunately, the hypothesis of [64] is based on the mistaken (and unsupported) idea that determinate growth necessarily involves cell expansion and fixed cell numbers among adults having different body sizes, whereas indeterminate growth entails cell multiplication and fixed cell size. The use of these terms in [60] does not follow the conventional definitions, which are that determinate growth ceases at sexual maturation, whereas indeterminate growth continues after maturation [236,237]. These modes of growth do not require specific patterns of cell growth or multiplication.

**Figure 5:**
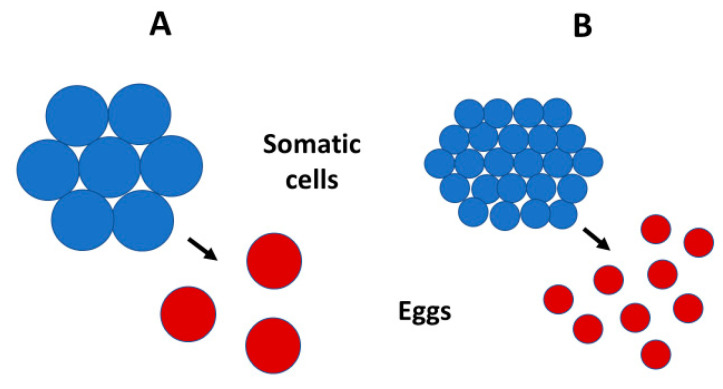
Schematic diagrams showing how the size and number of somatic cells (blue circles) in multicellular organisms tend to parallel the size and number of reproductive propagules (here illustrated as eggs: red circles), following prediction #5 of the Single-Cell ‘Bottleneck’ Hypothesis (SCBH: Table 6). (**A**): An organism with relatively few large somatic cells produces relatively few large eggs. (**B**): An organism with relatively many small somatic cells produces relatively many small eggs. These differences are similarly produced by changes in genome size (see Table A2) and ambient temperature (see Section 4.6).

Prediction #5 (as illustrated in Figure 5; and following from assumptions 2–4 and 8) is supported by many observations that increased chromosome number (and thus DNA content per cell) is associated with not only increased cell size and reduced cell number, but also in parallel, increased propagule size and reduced propagule number. Numerous studies of polyploidy effects support this prediction especially well (see Section 4.5; and Table A2). Further evidence is provided by the striking contrast between copepods and decapods. In copepods, the interspecific scaling of egg mass is nearly isometric (slope near 1), whereas the scaling of egg number (clutch size) is not significantly different from 0 [101]. Conversely, in decapods, the interspecific scaling of egg mass is not significantly different from 0, whereas the scaling of egg number is nearly isometric [101] (see also Section 4.7). These patterns parallel the different interspecific variation in cell size and number in these two taxa. Variation in body size is more related to cell size than number in copepods, but more related to cell number than cell size in decapods, as already noted.

The SCBH is helpful in explaining much of the diversity of genome size in the living world, especially in relation to propagule size and adult body size, but other factors not considered here may also be influential. For example, the SCBH apparently cannot explain why genome size (DNA content per cell nucleus) is much larger in copepods and peracaridans than in cladocerans having equivalent body or egg masses (Figure 1C,D; see also [57]). Perhaps, the relatively small genome size of cladocerans is related to their relatively rapid growth rates ([57]; see also Section 4.7). I further evaluate the SCBH in Section 4.4, Section 4.5 and Section 4.6. In Section 4.7, I also use the SCBH to promote linking genomic theory to life-history and metabolic theory.

### 4.4. Relationships between the Sizes and Numbers of Somatic Cells and Those of Propagules or Gametes

#### 4.4.1. Data

Assumption #3 and prediction #5 (Figure 5) of the SCBH (Table 6) are supported by data in Table A1 and Table A2. Variation in the sizes of somatic cells parallels that of reproductive propagules or gametes (Table A1). Furthermore, increases in genome size (via genome duplication or polyploidy) usually result in congruous increases in the sizes of cells and reproductive propagules and decreases in their number (Table A2; see also Section 4.5). These similarities suggest that a common mechanism or set of mechanisms may underlie trade-offs between somatic cell size and number and between reproductive propagule size and number. This mechanism or set of mechanisms may involve functional relationships to genome size, at least in part, as discussed in Section 4.4.2 and Section 4.5.

#### 4.4.2. Hypothetical Nucleotypic Effects

Here, I discuss why interpopulation or interspecific variation in the sizes and number of somatic cells parallels that for reproductive propagules (following prediction #5 of the SCBH). My overall explanation has two key components: (1) genome size and cell size are tightly correlated (assumption #4 of the SCBH) and (2) during development, the genome of germ cells is transmitted to somatic cells of the body, thus causing parallel effects of genome size on the sizes of germ cells and somatic cells, and of multicellular reproductive propagules that are largely affected by variation in cell size (following assumptions #1, #2 and #3 of the SCBH). To understand these parallel effects, one must realize that DNA can affect phenotypes through not only informational transmission (‘genotypic effects’), but also non-informational, physical/mechanical, ‘nucleotypic effects’ (following [7,15,16,31,113,238,239,240,241]). Throughout my article, I use the phrase “nucleotypic effect” to refer to any effect of genome size on various cellular, physiological and life-history traits, which have been quantified in numerous experimental and correlation analyses that I cite. How nucleotypic effects work is not well understood and subject to considerable debate [7,8]. For further information, the reader should see the reviews in [7,8,16,174,176,183,194,241,242,243]. Suffice it to say here that, as a general rule, large cells appear to require larger genomes to support their greater structure and resource demands than do smaller cells. In effect, nucleotypic effects provide an explanation for assumption #4, a critical foundational piece of the SCBH.

**Figure 6:**
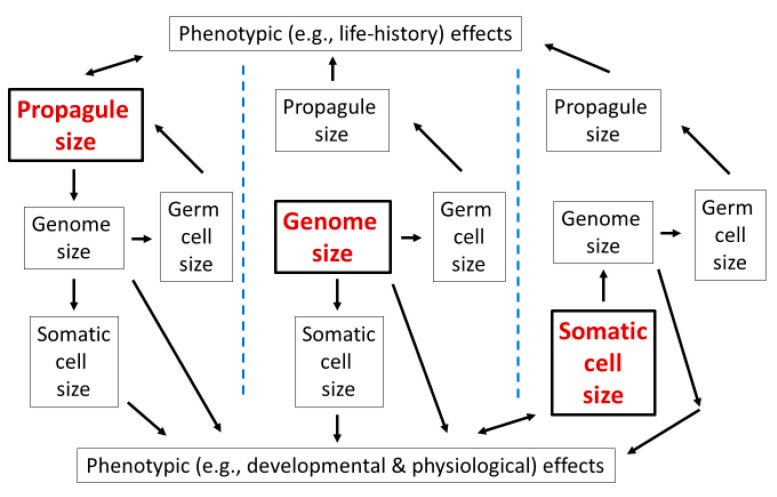
Hypothetical scenarios showing possible causal (functional or evolutionary) relationships among the sizes of reproductive propagules, genomes (DNA content per cell), somatic cells and germ cells. These scenarios, each of which may occur at least in some cases, attempt to explain why the sizes of the above entities are often positively correlated with one another (see Figure 1D, Figure 2D and Figure 4; Table 2, Table 3, Table 4 and Table 5, Table A1 and Table A2). The left-hand scenario hypothesizes that natural selection for larger reproductive propagules with relatively large cells favors larger genomes for structural and functional support. These larger genomes are then passed onto somatic cells and next-generation germ cells, which are also larger because of nucleotypic effects. The larger germ cells, in turn, contribute structurally and functionally to larger next-generation propagules, thus reinforcing the adaptive evolutionary effects. The selection for larger propagules may also be associated with changes in other life-history traits. In addition, changes in the sizes of somatic cells may have secondary effects on other phenotypic traits, including rates of growth, development and metabolism. The middle scenario hypothesizes that spontaneous or environmentally induced changes in genome size affect the sizes of somatic and germ cells, and secondarily propagule size and possibly other associated phenotypic traits. The right-hand scenario hypothesizes that natural selection for larger somatic cells favors larger genomes for structural and functional support. These larger genomes, in turn, support larger germ cells and reproductive propagules with possible secondary effects on other life-history traits. The selection for larger somatic cells may be direct or the indirect result of selection on other associated phenotypic traits. All of the hypothetical scenarios include a single-celled developmental stage, and as such are informed by the Single-Cell ‘Bottleneck’ Hypothesis (SCBH) described in Table 6.

Another fundamental and controversial question is whether genome size determines cell size or vice versa [7,8,103,176,181,183,187]. Many studies assume implicitly or explicitly that genome size determines cell size. This view is well supported by experimental manipulations of genome size that cause correlated effects on cell size (see also Section 4.5). However, these short-term experiments focus on immediate phenotypic effects and do not consider the long-term coevolution of genome size and cell size, as seen in interspecific comparisons. During evolution, it is possible that selection may favor larger (or smaller) cells, which in turn require larger (or smaller) genomes for structural and functional support [7,15,20,31,176,181,244]. If so, the following hypothetical scenario (Figure 6), involving both the long-term evolution of reproductive propagule cell size and its effect on genome size, and the short-term ontogenetic effects of genome size on somatic cell size, may result. Specific (e.g., cold, dry, resource-poor or highly competitive) environments may favor organisms that produce larger eggs, sperm, spores or other multicellular reproductive propagules (pollen and seeds) composed of relatively large cells (see also Section 4.6 and Section 4.7; and [15,101,245,246,247,248,249,250,251]). These cells may in turn require larger genomes. These large genomes are then transmitted to somatic cells and next-generation germ cells, which are relatively large because of nucleotypic effects. In addition, because of spatial (body-volume) constraints (following assumption #8), organisms in these specific environments may produce larger, but fewer somatic cells and reproductive propagules than those with similar body sizes in other environments favoring smaller propagules (also see Figure 5). Other hypothetical possibilities involving selection on the sizes of somatic cells (or their correlates, such as rates of growth, development and metabolism [7,8,15,89,96,176,181,218,244,252,253,254,255,256,257,258]) with secondary effects on genome size and propagule size, or effects of spontaneous or environmentally induced duplication of DNA sequences or whole genomes [8,11,17,103,172,186,242] on the sizes of somatic cells and reproductive propagules (Figure 6) should also be considered and evaluated. Mechanisms underlying relationships among genome size, cell size and propagule size are likely complex and multidirectional in cause-and-effect (Figure 6; see also Section 4.7).

Of course, the hypothetical scenarios depicted in Figure 6 assume that genome size and cell size are not altered during the ontogenetic development of various cell lineages. However, in specific cases, genomes (and their cells) may be up- or down-sized in specific tissues (e.g., [7,8,11,45,103,185,224,258,259]). Nevertheless, frequently observed associations between the sizes of somatic cells and their genomes and that of germ cells and reproductive propagules (Table A1 and Table A2) suggest that the above cases are exceptions to a general rule. In short, unicellular bottlenecks in the life cycles of multicellular organisms may affect not only the genome composition of their somatic cells by minimizing the effects of somatic cell mutants on organismal genetic lineages [156,157,158], but also their genome sizes via nucleotypic effects.

### 4.5. Effects of Polyploidy on the Sizes and Numbers of Cells, Gametes and Propagules

Numerous studies have shown that the sizes and numbers of somatic cells and reproductive propagules often correlate with genome size (e.g., [8,9,87,150,195]; see also Table 5 and Section 4.3). These associations are most clearly shown by comparing the sizes and numbers of somatic cells and reproductive propagules to the level of polyploidy among individuals, populations or species of organisms. Numerous examples for unicellular organisms and multicellular plants and animals are listed in Table A2: increasing ploidy correlates with larger but fewer somatic cells in 90 reported cases, larger sizes of both somatic cells and reproductive propagules in 58 cases, and larger but fewer propagules in 21 cases. Very few deviations from these trends have been reported. Many of the cited studies involve inducing polyploidy experimentally (e.g., by colchicine treatments). These experiments are especially useful for providing insight into cause-and-effect relationships.

### 4.6. Temperature Effects on Sizes of Cells, Gametes and Propagules

Experiments may also be used to manipulate the sizes of cells and propagules directly, independently of genome size. Most of these studies involve testing whether the effects of temperature on body size relate to changes in the sizes of somatic cells, reproductive propagules, or offspring. A common finding in ectothermic organisms is that decreasing temperature is associated with not only larger adult body size (following the ‘temperature-size rule’ [260]), but also significantly larger cells, propagules and (or) offspring (e.g., [103,218,219,248,250,252,261,262,263,264,265,266,267,268,269,270,271,272,273,274,275,276,277,278,279,280,281,282,283,284,285,286,287,288,289]). These studies provide further evidence that the sizes of somatic cells and reproductive propagules tend to be positively correlated (as illustrated in Figure 5).

Moreover, a short-term experimental study on the fruit fly *Drosophila melanogaster* showed that lower temperatures induced the growth of larger cells and nuclei without any change in genome size [290] (though an experimental study on bacteria showed that warming caused decreases in both genome size and cell size [285]). Therefore, although cell size and genome size are usually strongly correlated, it is possible that cell size can change without changes in genome size (see also [132,203,291]). Genome size does not always determine cell size, thus opening up the possibility that cell (or propagule) size may first change and only later through evolution be accompanied by changes in genome size. Increases in both cell size and genome size (including polyploidy) along natural environmental gradients of decreasing temperature, as observed in various protists, plants and invertebrate animals (e.g., [9,56,61,62,103,182,191,192,276,280,292,293,294,295,296,297,298,299,300,301,302,303,304]; but see [90,118,172,183,193,305]), may be the result of long-term adaptive evolution. If so, they (in combination with the laboratory experiments of [290]) provide support for the hypothetical view described in Section 4.4.2 (Figure 6) that, on an evolutionary timescale, changes in cell size may precede changes in genome size (also see next Section 4.7).

### 4.7. Linking Genomics with Life-History and Metabolic Theory

#### 4.7.1. Linking Genomics with Life-History Theory

The findings of this study and arguments made in Section 4.3 and Section 4.4 suggest that an understanding of genome-size diversity would benefit from a life-history perspective, as pioneered by Cavalier-Smith [15]. He suggested that much of the variation of genome size could be explained in terms of the life-history theory of r- and K-selection [306] (see also [57,178,243,253,293,300,307]). According to this view, small genomes are associated with r-selected traits, such as high colonizing ability, rapid individual and population growth, early maturation, high reproductive output and short lives that are favored in unstable or ephemeral habitats and at low population densities, whereas large genomes are associated with K-selected traits, such as high competitive ability, slower individual and population growth, late maturation, low reproductive output and long lives that are favored in stable habitats and at high population densities. Although the theory of r- and K-selection may help explain some variation in genome sizes (e.g., the association of large genomes with relatively slow growth rates and long lives in some protists, plants and ectothermic animals (e.g., [7,8,15,19,59,60,113,151,177,186,189,195,243,255,293,300,308,309,310,311,312]; but not in endothermic vertebrates [95]), and the association of relatively large genomes with larger, but fewer reproductive propagules ([146,166,217,219,313,314]; Table A2), it cannot explain why genome size covaries with body size in some taxa, but not others (as observed in Table 1).

**Figure 7:**
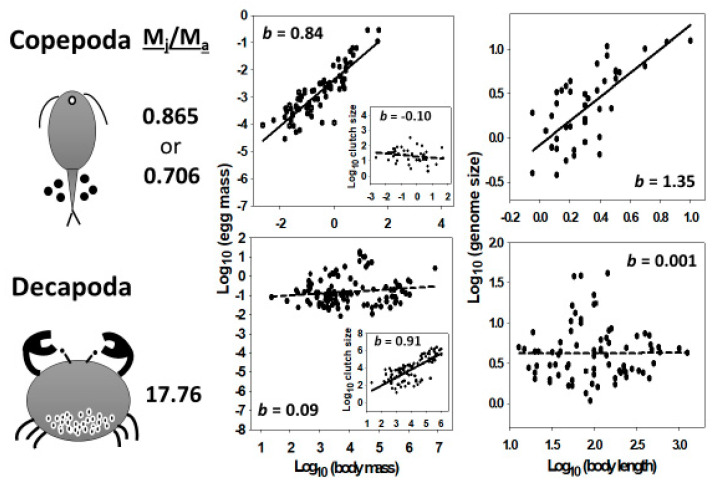
Body-mass scaling of egg mass and number per clutch (left-hand graphs) (data from [101]), and body-length scaling of genome size (right-hand graphs) (data from [57]) in copepods and decapods having different ratios of juvenile/adult mortality (M_J_/M_A_) (data from Table A3). For copepods, the top ratio is based on M_J_ for nauplii, whereas the bottom ratio is based on M_J_ for copepodids. The scaling exponent (slope, *b*) is indicated for each relationship. Hypothetical effects of M_J_/M_A_ on the observed scaling relationships are discussed in Section 4.7.1 (also see [101]).

I argue that additional life-history theory is needed to provide further insight into variation of genome size and its relationship to variation in body size and propagule size. In particular, life-history theory based on age- and size-specific mortality [237,245,315,316] may be especially useful in this respect. According to this theory, variation in juvenile mortality relative to adult mortality can have profound effects on life histories, including growth rates, the age and size at maturation, offspring size and number, and breeding frequency. For example, Glazier [101] has used this theory to explain why in copepods egg mass, but not egg number per clutch, strongly correlates with body mass, whereas in decapods the opposite occurs. He hypothesized that the ratio of juvenile/adult mortality (M_J_/M_A_) is relatively low in copepods, thus favoring increased investment in individual offspring at the expense of number as total reproductive investment associated with larger body sizes increases (total clutch mass scales isometrically with maternal body mass in crustaceans: [101]). In contrast, he hypothesized that M_J_/M_A_ is relatively high in decapods, thus favoring increased investment in number rather than size of offspring as total body-size related reproductive investment increases. When juvenile survival is relatively high and adult survival relatively low (and thus the probability of future reproduction is greater in juveniles than adults), the fitness of individual offspring (which relates to their energy stores and overall size) should be prioritized over parental fitness (which relates to both the size and number of offspring), thus favoring the allocation of increasing reproductive investment to larger, rather than more offspring, as observed in copepods. However, when juvenile survival is relatively low and adult survival relatively high (and thus the probability of future reproduction is greater in adults than juveniles), parental fitness should be prioritized over that of individual offspring, thus favoring the allocation of increasing reproductive investment to more, rather than larger offspring, as observed in decapods. Data shown in Figure 7 support this hypothesis. Copepods exhibit much lower M_J_/M_A_ than do decapods.

Following the SCBH, the above observations also help to explain why genome size scales positively with body size in copepods, but not in decapods (Figure 1A,C and Figure 7). Larger reproductive propagules with larger cells require larger genomes for structural and functional support. Therefore, genome size should also relate to M_J_/M_A_, at least indirectly.

Changes in genome size may not only result from life-history changes, but also cause them [103]. Variation in genome size is often (but not always) associated with changes in various life-history traits, including not only propagule size and number, but also growth rate, duration of developmental periods, and age at sexual maturity ([8,9,10,11,15,16,19,32,48,57,58,59,60,80,97,103,109,113,177,186,189,192,195,255,293,294,308,309,310,311,312,313,314,317]; see also sources cited in Table 2; but for contradictory evidence, see [97,98]). Interspecific correlations between genome size and longevity have also been proposed [48], but questioned [9,97]. Experimental manipulations of genome size (ploidy) provide critical evidence that genome size can affect life-history traits (e.g., [166,195]; also see sources cited in Table A2).

#### 4.7.2. Linking Genomics with Metabolic Theory

Metabolism fuels all biological activities, including key life-history processes such as growth and reproduction [318,319]. Furthermore, cell size may affect metabolic rate by means of surface area-to-volume effects. Surface-area-limited resource uptake and waste removal should scale to the 2/3 power of cell mass in isomorphic cells, whereas volume-related resource requirements should scale more steeply (log-log slope ≈ 1) with cell mass. Therefore, as cells grow, increasing limits on resource supply relative to resource-requiring cytoplasmic mass should cause them to have increasingly lower mass-specific metabolic rates. Maintaining ionic gradients is also less costly in larger cells with less surface area per volume. Therefore, an organism with few large cells should have a lower metabolic rate than an organism of similar size that has relatively many small cells [253,320,321]. In addition, the cell-size theory of metabolic scaling posits that if organisms grow by cell enlargement only, their total cell-surface-area and thus metabolic rate should scale to the 2/3-power of body mass. However, if they grow by cell multiplication only, their total cell-surface-area and thus metabolic rate should scale isometrically (log-log slope ≈ 1) with body mass. Or, if organisms grow by both cell enlargement and multiplication, the metabolic scaling exponent should be between 2/3 and 1 [205,320,321,322,323,324]. Consequently, if increasing genome size requires larger cells (following assumption #4 of the SCBH), then organisms with large genomes should also have lower mass-specific metabolic rates than those with smaller genomes [15,238,253].

The above genome-size hypothesis of metabolism has been tested many times with mixed results. As predicted, interspecific analyses often show that mass-specific metabolic rate is negatively related to genome size [8,118,205,238,253,256,257,320,325,326,327,328,329] (but see [330]). However, intraspecific tests in animals comparing polyploids with diploids have shown that increasing ploidy more often has no effect on metabolic rate than negative effects, and sometimes positive effects have even been observed (reviewed in [331,332,333,334]; also see [335,336]), as also seen for rates of photosynthesis in plants (e.g., [195,201,293,333]). Differences in metabolic rate between polypoid and diploid animals may be temperature-dependent [334,335,336]. In addition, some studies have shown that, although metabolic rate and its scaling with body mass relate to variation in cell size, they do not relate to variation in genome size [337,338]. Furthermore, although some intraspecific studies show relationships between cell size and metabolic scaling [321,322,323,339,340,341], others do not [342,343,344]. These results, suggest that interspecific associations between genome size and metabolic rate may be the result of the coevolution of genome size with cell size and metabolic rate, rather than direct effects of genome size on metabolic rate. Multiple cause-and-effect relationships may be involved, including selection for increased metabolic rate favoring the evolution of smaller cells and supporting genomes (see also Figure 6; and Section 4.4.2). The causes and consequences of the coevolution of genome size with various cellular, physiological and life-history traits are further discussed in the next Section 4.7.3.

#### 4.7.3. Genome Size as an Inter-Linking Component of Multi-Trait Adaptive Syndromes

Correlation analyses, as used this study, do not allow conclusive determination of cause-and-effect relationships. Incisive multivariate experimental and comparative analyses are needed to unravel the various causal pathways likely involved in relationships between genome size and reproductive propagule size, somatic cell size, body size, and various other phenotypic (developmental, physiological and life-history) traits (Figure 6). Artificial selection experiments may be especially valuable in this respect (e.g., [345]). Several investigators have emphasized that multiple causal pathways are likely involved in the evolution of genome size (e.g., [7,8,90,97,183,186,327]).

The life-history approach that I promote in this essay is only one of many possible multi-directional causal pathways involved in the evolution of genome size (Figure 6; see also Section 4.4.2). Nevertheless, it has three features that I believe make it especially worthy for further investigation.

First, it emphasizes the importance of the evolution of propagule size as a driving influence on genome size, cell size and other phenotypic traits (Figure 6; and Section 4.4.2), which has received little explicit consideration (though this view was intimated in [15,16]; also see [90]). As Bernardo [246] emphasized, phenotypes of eggs and other propagules relate to the genotypes and evolutionary fitness of both parents and offspring. I would add that they relate to the nucleotypes of both parents and offspring, as well (cf. [16]). Others have further argued that the egg is the most influential cell in an animal’s life history [346], and that its size strongly influences many other life-history traits [346,347,348]. Therefore, propagule size should be considered a key factor in a comprehensive understanding of the evolution of genome size and other associated phenotypic traits (also see Section 5).

Second, my approach helps to explain a greater congruity between the evolution of reproductive strategies and somatic cellular structure and function than has been hitherto appreciated (Figure 6). Nucleotypic and environmental factors that influence the size and number of somatic cells in a body usually have parallel effects on the size and number of reproductive propagules that are produced (Figure 5; also see Section 4.3, Section 4.4, Section 4.5 and Section 4.6; and Table A1 and Table A2). These parallel patterns are also supported by reports made over 100 years ago that in frogs and other animals relatively large gametes tend to give rise to adult bodies with relatively large somatic cells [261,349]. Unfortunately, these reports were largely ignored and forgotten, chiefly due to claims that they were not of general significance [208]. My analyses suggest that the pioneering findings of Chambers [261] and Popoff [349] were prematurely dismissed and deserve renewed attention.

Third, my approach emphasizes genome size as a critical connecting link between various reproductive and somatic traits (Figure 6; see also Section 5). For example, if selection favors larger (but fewer) somatic cells in the body, and thus larger supporting genomes, nucleotypic effects may, in turn, result in the production of larger (but fewer) propagules via enlargement of their cells. Alternatively, if selection favors larger (but fewer) propagules, larger supporting genomes may also be favored that, via nucleotypic effects, result in larger somatic cells. Or these causal pathways may both occur, resulting in an evolutionary or functional co-adjustment of the sizes of genomes, cells and propagules.

Multivariate, multidirectional approaches to genome-size evolution can be further understood in light of the ‘adaptive syndrome’ concept [350,351,352,353]. An adaptive syndrome is a “coordinated set of characteristics” (p. 139 in [350]) evolved in a specific ecological context (e.g., with respect to resource use, dispersal strategy, predator avoidance, survival in extreme environments, etc.). It recognizes that natural selection does not act on individual traits in isolation, but on constellations of phenotypic traits [354]. Although the ecological and behavioral aspects of adaptive syndromes have received some attention [351,352], their origin(s) is(are) little understood. According to traditional evolutionary theory, one may presume that natural selection acting on variable genes has driven the evolution of adaptive syndromes, perhaps in a step-wise gradual manner [355,356]. However, other kinds of mechanisms, including synergistic functional linkages and antagonistic trade-offs, and allometric, developmental, physiological and structural constraints may also be important in channeling, expediting or hindering evolution toward specific sets of phenotypic traits. This is a large topic that I cannot discuss fully here. Here, I would like to focus on the potentially important roles of nucleotypic effects and phenotypically plastic responses in facilitating or retarding the evolution of specific adaptive syndromes that involve the sizes of cells and genomes.

As previously emphasized, nucleotypic effects underpin how changes in genome size relate to a plethora of phenotypic changes, including changes in the size and number of somatic cells and reproductive propagules, and of the rates of growth, development and metabolism (see also Section 4.4.2). This “nucleotypic bond” [329] involves a cascade of synergistic phenotypic changes that may facilitate adaptation to specific kinds of environments because each phenotypic trait responds in a way that increases fitness. For example, in resource-poor and other kinds of stressful environments, increased sizes of cells and propagules and lower rates of growth, development and metabolism may all be advantageous responses (see, e.g., [101,253,300,309,357]). Perhaps this is why organisms with large genome sizes (including polyploids) often occur in stressful environments (e.g., [103,193,194,195,287,293,336,358,359]).

Similarly, phenotypically plastic responses to cold environments often involve increases in the sizes of somatic cells and reproductive propagules, and decreases in the rates of growth, development and metabolism, as well (see Section 4.6; and, e.g., [360,361,362,363,364]). These coordinated, multi-faceted phenotypic changes may be not only adaptive themselves (a point that is currently being debated [103,264,265,267,268,269,270,274,282,365,366]), but also the vanguard for further adaptive (genotypic) evolution in cold environments (or in an opposite way in hot environments). This view is in line with recent arguments that phenotypic plasticity is centrally important to the evolution of integrated phenotypic complexes (e.g., [354,367,368,369,370]). Coordinated phenotypic norms of reaction, as observed in plastic thermal responses, may often precede and facilitate the adaptive evolution of integrated phenotypes.

Therefore, both nucleotypic effects and phenotypically plastic responses may facilitate the coordinated evolution of adaptive syndromes in specific habitats. Additionally, propagule size may be an essential component of many of these adaptive syndromes (also see Section 5). However, some environments or life styles may favor the decoupling of genome size, cell size and various physiological and life-history traits. For example, comparisons of major taxa of crustaceans reveal that genome size and egg (propagule) size may be decoupled: at a given body size, cladocerans have much larger eggs, but much smaller genomes than do copepods (compare Figure 1A,C with Figure 1B). Why this is so deserves further investigation. In any case, it is possible that nucleotypic effects and phenotypically plastic responses may not only facilitate the evolution of adaptive syndromes in specific ecological contexts, but also hinder them in others that favor discordant responses of genome size, cell size, propagule size, etc.

## 5. Conclusions

In my essay, I have grappled with the long-standing mystery about why genome size shows highly variable (positive, absent and negative) relationships with body size (see Table 1). Four key observations that help unlock this mystery are (1) genome sizes usually relate more strongly to the structural size of the cells making up a multicellular organism than to the size of the whole body, (2) nearly all multicellular organisms have a single-celled developmental stage, (3) multicellular organisms grow by increasing cell size or number or both, and (4) genome size often shows no or even negative relationships with cell number. These and other observations are incorporated into a Single-Cell ‘Bottleneck’ Hypothesis (SCBH) that rests on eight well-verified assumptions that are used to infer five testable predictions (Table 6) for which there are considerable support (see Section 4.3). As a result of focused statistical analyses on four major taxa of crustaceans and broader surveys of other kinds of unicellular organisms and multicellular plants and animals, I reach the following major conclusions:

1. Genome size often relates more positively to reproductive propagule size than adult size (see Figure 1 and Table 1, Table 2, Table 3 and Table 5). This makes sense because propagules are either single-celled (e.g., eggs, sperm and spores) or consist of a relatively few cells (e.g., pollen and seeds) whose size often relate strongly to propagule size. Therefore, since genome size and cell size are usually strongly positively related, genome size should often relate positively to propagule size, as well. By contrast, multicellular body size relates to either cell size or number or both. This fact leads to the next conclusion.
2. Genome size relates more positively to the size of unicellular organisms or small multicellular organisms whose variation in size relates strongly to variation in cell size, than to the size of relatively large multicellular organisms whose variation in size relates chiefly to variation in cell number (illustrated in Figure 3). This conclusion is supported by ubiquitous positive relationships between genome size and body size observed in unicellular organisms, frequently positive relationships between genome size and body size observed in small multicellular organisms (e.g., flatworms, polychaete worms, mollusks, copepods, cladocerans, ostracods, amphipods, mites and ticks, and some rotifers and insects), and no or weakly positive or negative relationships with body size observed in relatively large organisms (e.g., decapods, fishes, tetrapods, ferns and angiosperms; see Figure 3 and Figure 4; and Table 1). This conclusion is also supported by the observation that genome size scales curvilinearly (concave downward) with body length or mass in crustaceans, with a positive relationship at the small end of the body-size range, and an absent or negative relationship at the large end of the body-size range (see Figure 2 and Table 4). However, why some small animal taxa (e.g., nematodes, rotifers, oligochaete worms, spiders and some insects) do not show positive relationships between genome size and body size (see Table 1) remains a mystery.
3. Organisms with larger genomes (e.g., polyploids) or that have been exposed to low temperatures during their development tend to show parallel increases in the sizes of their somatic cells and reproductive propagules, and parallel decreases in their number (see Figure 5 and Table A1 and Table A2). Changes in somatic cell size and number are, in turn, often related to changes in various developmental and physiological traits (e.g., rates of growth and metabolism). These patterns suggest that variation in reproductive strategies may be more intimately linked to variation in somatic cell size and function than has been hitherto appreciated. Adaptive or phenotypically plastic changes in reproductive traits may often covary with somatic traits, which should be considered in future theoretical models of life-history evolution and metabolic ecology.
4. DNA may influence phenotypes via not only informational (genotypic) effects, but also non-informational, structural or mechanical (nucleotypic) effects. Nucleotypic effects appear to play a central role in the network of cause-and effect relationships among genome size, cell size, propagule size and various other physiological and life-history traits (see Figure 6). Nucleotypic effects and thermally induced phenotypic plasticity may facilitate the evolution of ‘adaptive syndromes’ (integrated suites of traits, including the sizes of genomes, cells and propagules, and the rates of growth, development and metabolism) especially in hot, cold, resource-poor and other kinds of stressful environments.
5. I promote and further develop a life-history perspective to understanding the evolution of genome size and its relationship to body size. Genome size may be affected by not only r-, K-and adversity-selection, but also variation in age- and size-specific mortality—in particular, the relative mortality of juveniles (M_J_) and adults (M_A_) (see also Section 4.7.1 and Section 4.7.3). I hypothesize that in organisms where M_J_/M_A_ is low, propagule size, cell size and genome size should show strong positive scaling with body size (as observed in copepods), but in organisms where M_J_/M_A_ is high, propagule size, cell size and genome size should scale weakly with body size or not at all (as observed in decapods). Furthermore, because of trade-offs between the size and number of propagules and somatic cells, low M_J_/M_A_ should be associated with weak or absent scaling of propagule and cell number with body size (as observed in copepods), whereas high M_J_/M_A_ should be associated with strongly positive scaling of propagule and cell number with body size (as observed in decapods) (see Figure 7). Genome size may both affect and be affected by the evolution of various life-history traits [103]. I argue that propagule size and number are key (central) traits in this respect, a view that has not received the attention that it deserves. Propagule size relates not only to the genotypic fitness of both offspring and parents, but also to genome size, cell size and many other phenotypic traits, both directly and indirectly by nucleotypic effects (see Figure 6), and thus, to many kinds of internal (biological) and external (ecological) factors. As such, propagule size appears to be a ‘hub trait’ that is highly connected to many other traits [371,372] in adaptive syndromes (correlation networks) representing the multiple interfaces of the genotype, nucleotype, phenotype and ecotype.

## 6. Recommendations for Further Research

- Further testing of the SCBH is needed, including rigorous multivariate statistical analyses of the relationships among genome size, propagule size, cell size, body size, and various other phenotypic traits in diverse kinds of plants and animals at various taxonomic levels. These analyses would benefit from using phylogenetically informed methods, which have not been employed in the preliminary analyses of crustaceans presented in my article.
- Why genome size and body size are sometimes negatively correlated (Table 1) has not been addressed in my study, and deserves further investigation. Perhaps, negative relationships occur because larger size is sometimes associated with smaller (rather than larger) cells (and thus supporting genomes), a hypothesis that should be tested.
- Experiments involving manipulations of, or artificial selection on the sizes of genomes, cells, propagules and (or) adults are needed to identify and disentangle cause-and-effect relationships (including the mechanisms underlying nucleotypic effects).
- Further syntheses of genomic theory with life-history and metabolic scaling theory are likely to be worthwhile. For example, theory regarding the origin(s) of genome-size diversity would benefit from explicit inclusion of life-history theories regarding the evolution of propagule size and number, and of cell-size-based metabolic scaling theory. Life-history and metabolic scaling theory may also benefit from explicit inclusion of genome-size-related nucleotypic effects (e.g., [205]).
- Scaling analyses of genome size and many other traits have focused mostly on adult size as the independent variable. Analyses based on the sizes of immature ontogenetic stages (as done in the present study) may provide new insights. As Bonner [154] emphasized, it is important to study organisms in the context of their whole life cycles, not just as adults.

## Figures and Tables

**Table 1:** Positive (POS), negative (NEG) or nonsignificant (NO) relationships between genome size (total or haploid DNA content per cell nucleus, pg) and body size in various taxa of unicellular and multicellular organisms.

Taxon	Relationship	Source
**UNICELLULAR ORGANISMS**		
Prokaryotes and eukaryotes	POS	[19,20]
Planktonic bacteria	POS	[21]
*Escherichia coli*	POS	[22]
Algae (phytoplankton)	POS	[23,24,25]
*Dunaliella tertiolecta*	POS	[26]
Bacillariophyceae (diatoms)	POS	[27]
*Ditylum brightwellii*	POS	[28]
*Thalassiosira* species	POS	[29]
Dinoflagellata	POS	[30]
Protists	POS	[31]
Ciliophora	POS	[32,33]
*Stentor coeruleus*	POS ^1^	[34]
**MULTICELLULAR PLANTS**		
Polypodiopsida (ferns)	NO	[35]
Angiospermae	NEG	[10]
Herbaceous species	POS	[36]
Perennial species	NEG	[12]
*Acacia* species	NO	[37]
*Brassica rapa*	NO	[38]
*Lolium multiflorum*	POS	[39]
*Nicotiana* species	POS/NO ^2^	[40]
*Senecio* species	POS	[41]
*Vicia faba*	NEG	[42]
*Zea mays*	NEG	[43]
**MULTICELLULAR INVERTEBRATE ANIMALS**		
Platyhelminthes (flatworms)	POS	[44]
Nematoda (round worms)	NO	[45]
Rotifera (Monogononta)	NO	[46]
*Brachionus plicatilis*	POS/NO ^3^	[47]
Annelida (segmented worms)	POS	[48]
Oligochaeta	NO	[49]
Polychaeta	POS	[50]
Dorvilleidae		
*Ophryotrocha* species	POS/NO ^4^	[48,51]
Mollusca	POS	[52]
Gastropoda (snails)		
*Viviparus contectus*	POS	[53]
Arthropoda		
Arachnida	POS	[54]
Acari (mites and ticks)	POS	[55]
Araneae (spiders)	NO	[56]
Crustacea		
Cladocera	NO	[present study]
	POS	[57]
Copepoda	POS	[44,57,58,59,60,61,62] [present study]
Decapoda	NO	[57]
	NEG	[present study]
*Synalpheus* species	NO	[63]
Ostracoda	POS	[64]
Peracarida	? ^5^	[present study]
Amphipoda	POS	[57,65,66]
Hexapoda (insects)		
Blattodea (cockroaches and termites)	NO	[67]
Coleoptera (beetles)		
Chrysomelidae	NO	[68]
Coccinellidae	NO	[69]
Lampryidae	NO	[70,71]
Tenebrionidae	NO	[72]
*Phylan semicostatus*	NEG	[73]
*Pimelia* species	NO	[74]
*Tribolium* species	NO	[75]
Diptera		
Chironomidae (midges)	NO/POS	[76]
Culicidae (mosquitoes)		
*Aedes albopictus*	NO	[77]
Drosophilidae (fruit flies)	NO	[78]
	POS	[79]
*Drosophila melanogaster*	POS ^6^	[80]
Hymenoptera		
Apidae (bees)		
*Melipona* species	NO	[81]
Formicidae (ants)	NO	[82]
Hemiptera		
Aphidoidea (aphids)	NO	[83]
Coccoidea (scale insects)	POS	[67]
Lepidoptera (moths and butterflies)	NO	[84,85]
Arctiidae	NEG	[85]
Geometridae	POS	[85]
Noctuidae	NO	[85]
Odonata		
Anisoptera (dragonflies)	POS	[86]
Zygoptera (damselflies)	NEG	[86]
**MULTICELLULAR VERTEBRATE ANIMALS**		
Actinopterygii (ray-finned fishes)	NO	[87]
Cyprinidae	NO	[88]
Tetrapoda (4-legged vertebrates)	NO	[89]
Anura (frogs and toads)	NO	[90]
Pipidae	NO	[91]
Caudata (salamanders)	NO	[90,92]
	POS	[93]
Dinosauria		
Sauropoda	NO ^7^	[94]
Aves (birds)	POS	[95,96,97]
Mammalia	POS	[95,98]
Artiodactyla	NO	[95]
Carnivora	NO	[95]
Chiroptera (bats)	NO	[95]
	POS	[99]
Pteropodidae (megabats)	NO	[100]
	NO/POS ^3^	[99]
Primates	NO	[95]
Rodentia	POS	[95]

^1^ Ploidy level used as measure of genome size. ^2^ Positive for dry body mass, but no effect for stalk height at first flowering. ^3^ Positive relationship found for a Pearson’s product moment correlation analysis, but no significant relationship found for a phylogenetically informed analysis. ^4^ No significant relationships were found Pearson’s product moment correlation analyses, but a significantly positive relationship was found for a phylogenetically informed analysis. ^5^ A positive trend is seen (see Table 2, Figure 1C), but the sample size (*n* = 7) is too small for adequate analysis. ^6^ Body size estimated as pupal size. ^7^ Genome size inferred from osteocyte lacunae volumes.

**Table 2:** Statistical details for scaling relationships between log_10_-transformed values of genome size (GS, pg) versus body length (BL, mm) or wet body mass (BM, mg), wet egg mass (EM, mg) versus wet body mass, and genome size versus wet egg mass for each of four major crustacean taxa ^1^.

Relationship	Taxon	Slope ^2^	Intercept ^2^	*r* ^3^	*n* ^4^	*p* ^5^
GS vs. BL	Cladocera	0.444 (±0.155)	−0.680 (±0.073)	0.756	28	<0.00001
GS vs. BL	Copepoda	1.354 (±0.419)	−0.078 (±0.159)	0.709	44	<0.00001
GS vs. BL	Peracarida	1.291 (±1.029)	−1.091 (±1.423)	0.504	19	0.017
GS vs. BL	Decapoda	0.001 (±0.168)	0.623 (±0.345)	0.002	79	0.986
EM vs. BM	Cladocera	0.390 (±0.168)	−1.828 (±0.213)	0.777	18	0.00015
EM vs. BM	Copepoda	0.842 (±0.111)	−2.377 (±0.117)	0.871	75	<0.00001
EM vs. BM	Peracarida	0.639 (±0.123)	−1.972 (±0.204)	0.798	64	<0.00001
EM vs. BM	Decapoda	0.094 (±0.031)	−1.190 (±0.520)	0.145	105	0.141
GS vs. BM	Cladocera	0.039 (±0.098)	−0.562 (±0.133)	0.309	10	0.384
GS vs. BM	Copepoda	0.432 (±0.222)	0.861 (±0.196)	0.807	12	0.0015
GS vs. BM	Peracarida	0.367 (±0.502)	0.025 (±0.931)	0.643	7	0.119
GS vs. BM	Decapoda	−0.194 (±0.126)	1.487 (±0.552)	0.573	23	0.0043
GS vs. EM	Cladocera	0.179 (±0.152)	−0.211 (±0.339)	0.694	10	0.026
GS vs. EM	Copepoda	0.972 (±0.420)	3.330 (±1.182)	0.852	12	0.00043
GS vs. EM	Peracarida	0.541 (±1.242)	1.047 (±1.034)	0.448	7	0.314
GS vs. EM	Decapoda	0.273 (±0.219)	0.873 (±0.223)	0.493	23	0.017

^1^ Data from [57,101,102]. ^2^ 95% confidence intervals in parentheses. ^3^ Pearson’s product-moment correlation coefficient. ^4^ Sample size. ^5^ Probability that correlation is due to chance.

**Table 3:** General linear model (GLM) analyses for scaling relationships between log_10_-transformed values of genome size (pg) versus wet body mass (BM, mg) and wet egg mass (EM, mg) for each of four major crustacean taxa ^1^.

Taxon	N	BM Effect Coefficient	*p*	EM Effect Coefficient	*p*
Cladocera	10	−0.098	0.074	0.343	0.0089
Copepoda	12	0.203	0.140	0.643	0.040
Peracarida	7	0.515	0.258	−0.373	0.676
Decapoda	23	−0.181	0.0025	0.247	0.0090

^1^ Data from [57,101,102].

**Table 4:** Statistical details for linear and curvilinear (polynomial, quadratic) scaling relationships between log_10_-transformed values of genome size (pg) versus body length (mm) or body mass (mg), egg mass (mg) versus body mass, and genome size versus egg mass in crustaceans ^1^.

Relationship	Y Intercept	X Term	X^2^ Term	r ^2^	*n* ^3^	*p* ^4^
GS vs. BL (linear)	−0.052	0.344		0.534	170	<0.00001
GS vs. BL (curvilinear)	−1.252	0.903	−0.217	0.588	170	<0.00001 0.00013
EM vs. BM (linear)	−2.217	0.391		0.758	262	<0.00001
EM vs. BM (curvilinear)	−2.101	0.688	−0.079	0.831	262	<0.00001 <0.00001
GS vs. BM (linear)	0.213	0.110		0.461	52	0.00058
GS vs. BM (curvilinear)	0.377	0.331	−0.058	0.674	52	<0.00001 0.00002
GS vs. EM (linear)	0.793	0.257		0.439	52	0.00112
GS vs. EM (curvilinear)	0.889	0.528	0.094	0.474	52	0.0132 0.163 ^2^

^1^ Data from [57,101,102]. ^2^ Pearson’s product-moment correlation coefficient. ^3^ Sample size. ^4^ Probability that correlation is due to chance. A second *p* value refers to the X^2^ term, which indicates whether the curvilinear relationship is a significantly better fit than the linear relationship.

**Table 5:** Positive (POS), negative (NEG) or nonsignificant (NO) relationships between genome size and propagule or gamete size in various taxa of multicellular organisms.

Taxon	Propagule or Gamete	Relationship	Source
**PLANTS**			
Bryophyta (mosses)	Sperm	POS	[106]
Polypodiopsida (ferns)	Spore	POS	[35,107]
Gymnospermae	Pollen	NO	[108]
	Seed	POS	[109]
*Pinus* species	Seed	POS	[110,111,112]
Angiospermae	Pollen	NO/POS	[9,113,114,115,116]
	Seed	POS	[9,10,36,109,114,117,118]
Perennial herbs	Seed	POS	[12]
Geophytes	Seed	NO	[119]
*Acacia* species	Seed	NO	[37]
*Achillea* species	Seed	POS	[120]
*Aesculus* species	Seed	NO/POS ^1^	[121]
*Allium* species	Seed	POS	[9,113]
*Anacardium occidentale*	Seed	POS	[122]
*Armeria maritima*	Pollen	POS	[123]
*Bouteloua curtipendula*	Pollen	POS ^2^	[124]
*Brassica rapa*	Seed	NO	[38]
*Cicer* species	Seed	POS	[125]
*Corchorus olitorius*	Seed	NO/POS ^3^	[126]
*Crepis* species	Pollen	POS	[127]
	Seed	POS	[127]
*Dasypyrum villosum*	Seed	POS	[128]
*Glycine max*	Seed	POS	[129]
*Gossypium* species	Pollen	POS	[130]
*Hemerocallis* varieties	Pollen	POS	[131]
*Hyacinthus orientalis*	Pollen	POS	[132]
*Hylocereus* species	Pollen	POS	[133,134]
	Seed	NO/POS/NEG ^4^	[133]
*Juglans rea*	Seed	POS	[135]
*Lavandula angustifolia*	Seed	POS	[136]
*Lolium multiflorum*	Seed	POS	[39]
*Lolium perenne*	Seed	POS	[137]
*Malus* × *domestica*	Pollen	POS	[138]
*Nicotiana* species	Seed	POS	[40]
*Pisum sativum*	Seed	POS	[139]
*Pyrus pyrifolia*	Pollen	POS	[140]
*Ramonda* species	Pollen	POS	[141]
*Ramonda* species	Seed	NO/POS ^5^	[141]
*Scilla sibirica*	Pollen	POS	[132]
*Senecio* species	Seed	NO	[41]
*Sisyrhinchium* species	Seed	POS	[142]
*Streptocarpus* species	Pollen	NO/POS ^6^	[143]
*Vicia* species	Seed	POS	[113,144]
*Vicia sativa*	Seed	POS	[145]
*Zea mays*	Seed	NEG	[43]
**INVERTEBRATE ANIMALS**			
Rotifera (Monogononta)	Egg	NO	[46]
*Brachionus plicatilis*	Egg	POS	[47]
Annelida (segmented worms)			
Oligochaeta			
Dorvilleidae			
*Ophryotrocha* species	Egg	NO	[48,51]
Mollusca			
*Crassostrea gigas*	Egg	POS	[146]
Arthropoda			
Crustacea			
Cladocera	Egg	POS	[present study]
Copepoda	Egg	POS	[present study]
Decapoda	Egg	POS	[present study]
Peracarida	Egg	? ^7^	[present study]
Insecta			
Coleoptera (beetles)			
Bruchinae	Egg	NO	[147]
Tenebrionidae			
*Tribolium* species	Sperm	POS	[75]
Diptera	Egg	? ^8^	[148]
Drosophilidae (fruit flies)	Sperm	POS	[79]
Drosophilidae (fruit flies)	Egg	NO	[79,149] my analysis
**VERTEBRATE ANIMALS**			
Actinopterygii (ray-finned fishes)	Egg	POS	[87,150]
Anura (frogs and toads)	Egg	NO	[90]
Pipidae	Egg	NO	[91]
Caudata (salamanders)	Egg	NO	[90]
Plethodontidae	Egg	POS	[151] my analysis
Mammalia	Sperm	NO/POS ^9^	[7,152,153]
Chiroptera	Neonate	NO	[99]

^1^ No significant relationship overall, but positive relationships within clades. ^2^ Chromosome number (ploidy) used as an indicator of genome size. ^3^ Significantly positive effect on seed surface area, but not for seed mass, length or width. ^4^ Associations varied with various diploid-tetraploid lines. ^5^ Weakly positive effect on mass, but not significantly different in structural size. ^6^ Positive correlation in polyploids, but not diploids. ^7^ An apparent positive trend (see Figure 1D; Table 2), but sample size (*n* = 7) is too small for adequate analysis. ^8^ Sample size (*n* = 5) is too small for adequate analysis, but two species with tiny eggs have very small genome sizes. ^9^ Positive associations with ploidy in rodents, but lack of correlation for general phylogenetically informed analyses.

**Table 6:** The eight assumptions and five predictions of the Single-Cell “Bottleneck’ Hypothesis (SCBH).

Assumption/Prediction	Statement
Assumption #1	The life cycles of most multicellular organisms include a single-celled developmental stage connecting one generation to the next.
Assumption #2	Reproductive propagules or gametes are unicellular (e.g., eggs/oocytes, sperm and spores) or consist of relatively few cells (pollen and seeds) compared to that of adults.
Assumption #3	Variation in the sizes of multicellular reproductive propagules is usually related to variation in cell size, at least in part.
Assumption #4	Genome size is almost always positively correlated with cell size.
Assumption #5	Genome size is usually unrelated or even negatively related to cell number in multicellular organisms.
Assumption #6	Multicellular bodies grow by cell enlargement or multiplication, or both.
Assumption #7	Large organisms typically require more cell multiplication to reach adult size than do small organisms, especially if the size differences are large.
Assumption #8	Trade-offs between somatic cell size and number and between propagule size and number often occur because of spatial (body-volume) constraints.
Prediction #1	Genome size should be more positively correlated with propagule size than adult body size. This prediction should apply to both unicellular and multicellular propagules.
Prediction #2	Genome size should be more strongly related to the size of a living system if it is unicellular than if it is multicellular.
Prediction #3	Genome size should be more strongly related to adult body size in multicellular organisms that differ mainly in cell size rather than cell number.
Prediction #4	Genome size should be more related to the size of a multicellular living system if it is small and chiefly affected by cell size (e.g., reproductive propagules and small adults) than if it is large and chiefly affected by cell number (e.g., large adults).
Prediction #5	Spatial (body-volume) constraints and similar effects of genome size on the sizes of somatic cells and reproductive propagules should cause interpopulation or interspecific variation in propagule size and number to parallel variation in somatic cell size and number.

## Data Availability

Not applicable.

## Supplementary Materials

The following are available online at https://www.mdpi.com/article/10.3390/biology10040270/s1, Table S1 Crustacean data on body mass, egg mass and genome size.

## Appendix A

Table A1 and Table A2 provide ancillary information that helps support arguments made in Section 4.3, Section 4.4, Section 4.5, Section 4.7 and Section 5.

**Table A1:** Studies showing significant positive associations between sizes of various types of reproductive propagules or gametes and sizes of somatic cells in various taxa of plants and animals.

Taxon	Propagule/Gamete 1	Propagule/Gamete 2	Cell Type 1	Cell Type 2	Source
**PLANTS**					
Bryophyta (mosses)					
*Octoblepharurn albidum*	Spore		Leaf		[373]
Polypodiopsida (ferns)	Spore		Stomata		[35]
*Dryopteris filix-mas*-Gruppe	Spore		Stomata		[374]
Angiospermae	Pollen	Seed	Stomata		[166]
*Allium oleraceum*	Pollen		Stomata		[375]
*Arabidopsis thaliana*	Seed		Embryo	Seed coat	[168,169]
	Seed		Stomata	Leaf epidermis	
*Brassica campestris*	Pollen		Stomata		[376]
*B. rapa*	Pollen	Seed	Stomata		[377]
*Bromus inermis*	Pollen		Stomata		[378]
*Catharanthus roseus*	Pollen	Seed	Stomata		[379,380]
*Chamomilla recutita*	Pollen	Seed	Stomata		[381]
*Convolvulus pluricaulis*	Pollen	Seed	Stomata	Leaf epidermis ^1^	[382]
*Cyamopsis psoraloides*	Pollen		Stomata		[383]
*Cyclamen persicum*	Pollen		Stomata		[384]
*Dactylis glomerata*	Seed		Stomata		[385]
*Echinacea purpurea*	Pollen	Seed	Stomata		[386]
*Eriotheca* species	Pollen		Stomata		[387]
*Fagopyrum tataricum*	Pollen	Seed			[388]
*Glycine max*	Pollen	Seed	Stomata		[389,390]
*Hemerocallis* varieties	Pollen		Stomata		[131]
*Hemerocallis flava*	Pollen		Stomata		[391]
*Hylocereus* species	Pollen	Seed ^2^	Stomata		[134]
*Hyoscyamus muticus*	Seed		Stomata		[392]
*Jatropha curcas*	Pollen	Seed	Stomata		[393]
*Lactuca sativa*	Pollen	Seed	Stomata		[394]
*Lagerstroemia indica*	Pollen	Seed	Stomata		[395]
*Lathyrus sativus*	Pollen	Seed	Stomata		[396]
*Lavandula angustifolia*		Seed	Stomata		[136]
*Lepidium sativum*	Seed		Stomata		[397]
*Linum* species	Pollen	Seed	Stomata		[398]
*Lolium multiflorum*	Seed		Stomata		[39]
*Lolium perenne*	Seed		Leaf epidermis		[137]
*Malus* × *domestica*	Pollen		Stomata		[138]
*Miscanthus* species	Pollen		Stomata		[399]
*Nicotiana* species		Seed	Stomata	Leaf epidermis	[40]
*Nigella sativa*	Seed		Stomata		[400]
*Ocimum basilicum*	Pollen		Stomata		[401]
*Oryza sativa*	Seed		Spikelet hull epidermis		[402,403]
*Phaseolus vulgaris*	Pollen	Seed	Cotyledon	Stomata ^3^	[167,216,404]
*Phlox amabilis*	Pollen		Stomata		[405]
*Physalis* species	Pollen		Stomata		[406]
*Pisum sativum*	Seed		Cotyledon		[139]
*Plantago media*	Pollen	Seed	Stomata		[407]
*P. ovata*	Pollen	Seed	Stomata		[408]
*P. psyllium*	Pollen	Seed	Stomata		[409]
*Pyrus pyrifolia*	Pollen		Stomata		[140]
*Raphanus sativus*	Pollen		Stomata		[410]
*Rhipsalis baccifera*	Seed		Stomata		[411]
*Sesamum indicum*	Pollen		Stomata		[412]
*Tanacetum parthenium*	Pollen	Seed	Stomata	Root meristem	[413]
*Trachyspermum ammi*	Pollen	Seed	Stomata		[414,415]
*Trifolium* species	Pollen		Stomata		[416]
*Vicia* species	Seed		Cotyledon		[144]
*Vicia villosa*	Pollen		Stomata		[417]
*Vigna* species	Pollen	Seed	Stomata		[418]
*Viola* × *wittrockiana*	Pollen	Seed			[419]
*Ziziphus jujuba*	Pollen		Stomata		[420]
**INVERTEBRATE ANIMALS**					
Arthropoda					
Insecta					
*Bombyx mori*	Egg		Serosa		[421]
**VERTEBRATE ANIMALS**					
Actinopterygii (ray-finned fishes)					
*Cobitus*	Egg		Erythrocyte		[314]
*Misgurnus anguillicaudatus*	Egg	Sperm			[422]
Anura (frogs)	Egg		Gastrula		[423]
*Rana* species	Egg		Epidermis	Lens ^4^	[261]
Mammalia					
Rodentia	Sperm		Liver		[153]

^1^ Additionally, leaf palisade cells. ^2^ Seed mass is positively or negatively associated with sizes of pollen and stomatal cells. ^3^ Additionally, hypocotyl and root endodermis cells. ^4^ Additionally, cartilage, muscle, rectum and other cell types.

**Table A2:** Effects of polyploidy on cell or propagule (or gamete) size and number in various taxa of unicellular and multicellular organisms.

Taxon	Cell Size	Cell Number	Propagule Size	Propagule Number	Source
**UNICELLULAR ORGANISMS**					
Prokaryotes	POS				[424,425]
Fungi					
*Saccharomyces cerevisiae*	POS				[426,427,428]
Bacillariophyceae (diatoms)					
*Thalassiosira* species	POS				[29]
Ciliophora					
*Stentor coeruleus*	POS				[34]
**MULTICELLULAR ORGANISMS**					
**PLANTS**					
Bryophyta (mosses)					
*Bryum* varieties	POS				[429]
*Octoblepharum albidum*	POS		POS		[373]
Polypodiopsida (ferns)	POS		POS		[107,430]
*Asplenium* species	POS				[431]
*Asplenium trichomanes* x *viride*-Bastarde	POS				[432]
*Dryopteris margina*	POS				[433]
*Dryopteris filix-mas*-Gruppe	POS		POS		[374]
*Woodwardia virginica*	POS				[433]
Angiospermae	POS		POS	NEG	[113,166,172,189,434]
*Abelmoschus* species	POS	NEG			[435]
*Acacia mearnsii*	POS	NEG			[436]
*Actinidia deliciosa*	POS				[437]
*Andropogon* species			POS		[438]
*Aegilops neglecta*	POS	NEG			[439]
*Allium oleraceum*	POS	NEG	POS		[375]
*A. sativum*	POS	NEG			[440]
*Anthurium andraeanum*	POS	NEG			[441]
*Arabidopsis thaliana*	POS		POS		[169,442,443,444,445]
*Arachis* species			POS		[446]
*Asparagus officinalis*	POS	NEG			[447]
*Atriplex confertifolia*	POS	NEG			[201]
*Averrhoa carambola*			POS		[448]
*Bletilla striata*	POS				[449]
*Brachiaria ruziziensis*	POS				[450]
*Brassica campestris*	POS	NEG	POS		[376]
*B. oleracea*			POS		[451]
*B. rapa*	POS		POS		[377]
*Bromus inermis*	POS	NEG	POS		[378]
*Buddleja macrostachya*	POS	NEG			[452]
*Calendula officinalis*	POS	NEG			[453]
*Camellia sinensis*	POS	NEG			[454]
*Cannabis sativa*	POS	NEG			[455]
*Carthamus tinctorius*	POS				[456]
*Catharanthus roseus*	POS	NEG	POS		[379,380]
*Cattleya intermedia*	POS	NEG			[457]
*Centella asiatica*	POS				[458]
*Chaenomeles japonica*	POS				[459]
*Chamerion (Epilobium) angustifolium*	POS	NEG	POS		[460,461]
*Chamomilla recutita*	POS		POS		[381]
*Chrysanthemum carinatum*			POS		[462]
*Chrysanthemum* (*Dendranthema × grandiflorum*)	NO		NO		[463]
*Citrulus lanatus*			POS	NEG	[464]
*Citrus clementine*	POS	NEG			[465]
*C. limonia*	POS				[466]
*C. reticulata*	POS	NEG			[467]
*Clematis heracleifolia*	POS	NEG			[468]
*Coffea species*	POS	NEG			[469]
*Convolvulus pluricaulis*	POS	NEG	POS	NEG	[382]
*Crataegus* species	POS				[470]
*Cyamopsis psoraloides*	POS	NEG	POS	NEG	[383]
*Cyclamen persicum*	POS		POS		[384]
*Cynodon dactylon*	POS	NEG			[471]
*Dactylis glomerata*	POS		POS	NEG	[385,472]
*Datura stramonium*	POS	NEG			[473]
*Dendrobium cariniferum*	POS	NEG			[474]
*Dioscorea zingiberensis*	POS				[475]
*Dracocephalum kotschyi*	POS				[476]
*Echeveria* ‘peerless’	POS	NEG			[477]
*Echinacea purpurea*	POS	NEG	POS		[386]
*Eragrostis curvula*	POS				[478]
*Eriotheca* species	POS		POS		[387]
*Fagopyrum tataricum*			POS		[388]
*Festuca arundinacea*	POS	NEG			[479]
*Fragaria vesca*	POS	NEG			[480]
*Gerbera jamesonii*	POS	NEG			[481]
*Glycine max*	POS	NEG	POS	NEG	[389,390]
*Glycyrrhiza glabra*	POS				[456]
*Hemerocallis* varieties	POS		POS		[131]
*Hemerocallis flava*	POS		POS		[391]
*Hibiscus syriacus*	POS	NEG			[482]
*Hordeum vulgare*	POS				[483]
*Humulus lupulus*	POS				[484]
*Hylocereus* species			POS/NO ^1^	NEG ^1^	[133]
*Hylocereus* species	POS	NEG	POS/NEG ^2^	NEG ^2^	[134]
*Hyoscyamus muticus*	POS		POS		[392]
*Impatiens balsamina*			POS	NEG	[485]
*Isatis indigotica*	POS		POS		[486]
*Jatropha curcas*	POS	NEG	POS/NEG ^3^		[393]
*Lactuca sativa*	POS		POS		[394]
*Lagerstroemia indica*	POS	NEG	POS		[395,487]
*Lathyrus sativus*	POS	NEG	POS	NEG	[396]
*Lavandula angustifolia*	POS		POS		[136]
*Lepidium sativum*	POS	NEG	POS		[397]
*Lilium davidii*	POS	NEG			[488]
*Linum* species	POS		POS		[398]
*Lobularia maritima*	POS	NEG			[489]
*Lolium* species	POS				[490]
*Lolium multiflorum*	POS		POS		[39,491]
*L. perenne*	POS				[491]
*Lycium ruthenicum*	POS	NEG			[492]
*Malus* × *domestica*	POS		POS		[138]
*Mentha canadensis*	POS	NEG			[493]
*Medicago sativa*	POS	NEG			[494]
*Miscanthus* species	POS		POS		[399,495]
*Morus alba*	POS	NEG			[496]
*Musa* species	POS	NEG			[497]
*Musa acuminata*	POS	NEG			[498]
*Nicotiana* species	POS	NEG	POS		[40]
*Nigella sativa*	POS		POS		[400]
*Ocimum basilicum*	POS	NEG	POS		[401]
*O. kilimandscharicum*	POS	NEG			[499]
*Onosma* species			POS		[500]
*Opuntia mesacantha*	POS				[501]
*Oryza sativa*			POS		[502]
*Paeonia varieties*			POS		[503]
*Papaver bracteatum*	POS	NEG			[504]
*Paulownia tomentosa*	POS	NEG			[505]
*Pennisetum* species	POS	NEG			[506]
*Petroselinum crispum*	POS	NEG			[507]
*Phaseolus vulgaris*	POS	NEG	POS		[404]
*Phleum* species	POS				[508]
*Phlox amabilis*	POS		POS		[405]
*Physalis* species	POS		POS		[406]
*Pinellia ternate*	POS	NEG			[509]
*Plantago media*	POS		POS	NEG	[407]
*P. ovata*	POS		POS	POS	[408]
*P. psyllium*	POS	NEG	POS		[409]
*Platanus acerifolia*	POS	NEG			[510]
*Plumbago auricalata*	POS	NEG			[511]
*Pogostemon cablin*	POS	NEG			[512]
*Poncirus trifoliata*	POS				[513]
*Populus* varieties	POS				[514]
*Populus tremuloides*	POS				[305]
*Primula sieboldii*			POS		[515]
*Pyrus pyrifolia*	POS		POS	NEG	[140]
*Ramonda* species			POS		[141]
*Raphanus sativus*	POS		POS		[410,516]
*Rhododendron fortunei*	POS	NEG			[517]
*Ricinus communis*	POS		POS		[518]
*Robinia pseudoacacia*	POS	NEG			[519]
*Salix* species	POS				[520]
*Salix viminalis*	POS				[521]
*Salvia officinalis*	POS	NEG			[522]
*Secale cereale*, *Triticum aestivum* and hybrids	POS	NEG			[523]
*Sesamum indicum*	POS	NEG	POS		[412]
Solanaceae			POS		[524]
*Setaria italica*			POS	NEG	[525]
*Solanum phurela*	POS				[526]
*Sorghum bicolor*	POS	NEG			[527,528]
*Spathiphylum walisii*	POS	NEG			[529]
*Tagetes erecta*	POS	NEG			[530,531]
*Tanacetum parthenium*	POS	NEG	POS		[413]
*Taraxacum* species	POS	NO			[532]
*Thalictrum alpinum*	POS	NEG			[533]
*Themeda triandra*			POS	NO/POS ^4^	[534]
*Thymus persicus*	POS	NEG			[535]
*Tradescantia canaliculata*	POS	NEG			[190]
*Trachyspermum ammi*	POS	NEG	POS	NEG	[414,415]
*Trichosanthes dioica*				NEG	[536]
*Trifolium* species	POS		POS		[416]
*Tripleurospermum* species	POS				[537]
*Triticum* species	POS	NEG	POS		[538,539]
*Vanilla planifolia*	POS				[540]
*Viburnum* species	POS	NEG			[541]
*Vicia cracca*			POS		[542]
*V. faba*	POS	NEG			[543]
*V. villosa*	POS	NEG	POS		[417]
*Vigna* species	POS	NEG	POS		[418]
*Viola* × *wittrockiana*			POS	NEG	[419]
*Zantedeschia* varieties	POS				[544]
*Zea mays*	POS				[545]
*Zingiber officinale*	POS				[546]
*Ziziphus jujuba*	POS	NEG	POS		[420,547]
**INVERTEBRATE ANIMALS**					
Mollusca					
Bivalvia					
*Crassostrea gigas*			POS	NEG	[146]
*Mulinia lateralis*			POS	NEG	[217]
Gastropoda					
*Bulinus*			POS		[548]
*Potamopyrgus antipodarum*			POS		[549]
Arthropoda					
Crustacea					
Anostraca					
*Artemia parthenogenetica*				NEG	[550]
*A. salina*	POS	NO/NEG^5^			[551]
Cladocera					
*Daphnia pulex* complex			POS	NEG	[218,313]
Decapoda					
*Penaeus chinensis*	POS	NEG			[204]
Insecta					
*Bombyx mori*	POS	NEG	POS		[421,552]
**VERTEBRATE ANIMALS**					
Actinopterygii (ray-finned fishes)	POS				[553,554]
*Acipenser baeri*	POS				[555]
*Carassius auratus*	POS				[556,557]
*C. gibelio*			POS	NEG	[558]
*Cobitus* species	POS		POS	NEG	[222,314]
*Cobitis biwae*	POS				[559]
*Ctenopharyngodon idella* × *Hypophthalmichthys nobilis* hybrids	POS				[560]
*Cyprinus carpio*	POS	NEG			[561]
*Danio rerio*	POS	NEG			[562,563]
*Dicentrarchus labrax*			POS		[564]
*Gasterosteus aculeatus*	POS	NEG			[199]
*Ictalurus punctatus*	POS				[565]
*Misgurnus anguillicaudatus*			POS		[422]
*M. fossilis*			POS		[566]
*M. mizolepis*	POS				[567]
*Oncorhynchus kisutch*	POS	NEG	POS		[568,569]
*O. mykiss*	POS	NEG			[570,571]
*Oreochromis* varieties	POS				[572]
*Oreochromis aureus*	POS				[573]
*Plecoglossus altivelis*	POS	NEG			[574]
*Pleuronectes platessa*			POS	NEG	[575,576]
*Poeciliopsis* species	POS				[577]
*Pomoxis annularis*	POS				[578]
*Rhodeus ocellatus*			POS	NEG	[579]
*Salmo gairdneri*			POS		[580]
*S. salar*	POS	NEG			[568,581]
*S. trutta*	POS				[582]
*Salvelinus fontinalis*	POS				[583]
*Stizostedion varieties*	POS				[584]
*Tilapia aurea*	POS				[585]
*Tinca tinca*			POS	NEG	[586]
Anura (frogs)					
*Bufo viridis* complex	POS				[587]
*Hyla* species	POS				[588]
*Hyla versicolor* complex	POS		POS		[589,590]
*Neobatrachus* species	POS				[200]
*Odontophrynus* species	POS				[591]
*Odontophrynus americanus*	POS				[592]
*Pleurodema* species	POS				[591]
*Pelophylax* (*Rana*) species	POS				[593]
*Pelophylax esculentus*	POS				[284]
*Xenopus laevis*	POS				[594]
Caudata (salamanders)					
*Ambystoma* species	POS			NEG	[595]
*Ambystoma jeffersonianum* complex	POS				[596]
*Ambystoma laterale-texanum* hybrid complex			POS		[347]
*Triturus viridescens*	POS	NEG			[196,597]
Mammalia					
Rodentia			POS		[153]
*Mus musculus*	POS	NEG			[197,198,202,258]

^1^ Increased ploidy is associated with larger pollen, and fewer seeds of similar size. ^2^ Increased ploidy is associated with larger pollen and fewer seeds with either higher or lower mass. ^3^ Increased ploidy is associated with larger pollen and seeds having greater structural size, but lower mass. ^4^ Effect of ploidy on seed production depends on temperature and moisture. ^5^ Effect of ploidy on cell number depends on tissue type.

## Appendix B

Table A3 presents data used to calculate the mean ratios of juvenile mortality relative to adult mortality (M_J_/M_A_) in copepod and decapod crustaceans, as depicted in Figure 7. The M_J_/M_A_ ratios were calculated by dividing the average M_J_ by the average M_A_ for each taxonomic group. Sample sizes for nauplii, copepodids, adult copepods, larval decapods and adult decapods are 19, 10, 12, 5, and 21, respectively.

**Table A3:** Instantaneous natural mortality rates (d^−1^) ^1^ of larval juveniles (M_J_) and adults (M_A_) of copepod and decapod crustaceans.

Species	M_J_	M_A_	Source
**COPEPODA**			
*Acartia clausi*	0.2243 (N)		[598]
*A. hudsonii*		0.063	[599]
*A. tonsa*	0.7606 (N)	0.6	[598,599,600]
*Calanus glacialis*	0.11 (C)		[601]
*C. finmarchicus*	0.13 (N)	0.102	[602,603,604,605,606]
	0.097 (C)		
*C. helgolandicus*	0.426 (N)	0.1175	[598,602,607]
*C. pacificus*		0.065	[608]
*C.* spp.	0.0975 (N)		[609]
	0.052 (C)		
*Centropages typicus*	0.2398 (N)		[598]
*Clausocalanus furcatus*	1.0165 (N)	0.485	[603]
	0.314 (C)		
*Diaptomus clavipes*	0.365 (N)	0.23	[603]
	0.014 (C)		
*D. negrensis*	0.53 (N)	0.80	[603]
	0.878 (C)		
*Eurytemora affinus*	1.01 (N)	0.265	[598,599,600]
*Euterpina acutifrons*	0.2322 (N)		[598]
*Oithona amazonica*	0.11 (N)	1.2	[603]
	0.844 (C)		
*O. helolandica*	0.1233 (N)		[598]
*O. nana*	0.0399 (N)		[598]
*O. similis*	0.0194 (N)	0.0718	[601,603,609,610]
	0.02 (C)		
*Paracalanus parvus*	0.0874 (N)		[598]
*Pseudocalanus elongatus*	0.04 (N)		[611]
	0.03 (C)		
*P. newmani*	0.11 (N)	0.0965	[612,613]
*P.* sp.	0.05 (N)		[600]
	0.05 (C)		
**DECAPODA**			
(Shrimp)			
*Acetes japonicas*		0.00644	[614]
*Crangon crangon*		0.00945	[615,616]
*Litopeneaus schmitti*		0.00662	[617]
*Macrobrachium equidens*		0.00737	[618]
*M. macrobrachion*		0.0092	[619]
*M. völlenhovenii*		0.00764	[620,621]
*Palaemon adspersus*		0.00593	[622]
*Pandalus jordani*	0.04865 (Z)	0.00436	[600,623,624]
*P. borealis*		0.00253	[625,626]
*Penaeus duorarum*	0.22 (Z)		[600]
*P. latisulcatus*		0.00386	[627,628]
*P. semisulcatus*		0.00658	[629]
(Lobsters)			
*Panulirus interruptus*	0.018 (Z)		[600]
*P. penicillatus*		0.000986	[630]
(Crayfish)			
*Astacus leptodactylus*		0.00158	[631]
(Crabs)			
*Callinectes sapidus*		0.00240	[632]
*Cancer magister*	0.0161 (Z)	0.00440	[600,633,634]
*C. pagurus*		0.00155	[635]
*Chionoecetes bairdi*		0.000562	[636]
*C. opilio*		0.00146	[636,637,638]
*Lithodes aequispinus*		0.00145	[639]
*Pagurus* spp.	0.062 (L)		[640]
*Paralithodes camptschaticus*		0.00140	[639,641]
*P. platypus*		0.000515	[639]

^1^ Instantaneous (daily) natural mortality rates (M) were calculated typically as M = ln(N_0_/N_t_)/-t, where N_0_ is the initial number of individuals in a cohort and N_t_ is the number of surviving individuals after the time interval t in days (e.g., [600]). These rates excluded effects of human harvesting. Although mortality rates were estimated at various temperatures and other environmental conditions, major differences of M_J_/M_A_ between copepods and decapods are apparent. Averages were calculated for species with multiple values. N = nauplii. C = copepodids. Z = zoea. L = larvae.
